# Supplementary material for: Molecular diagnosis of Trichuris trichiura: Prevalence and associated risk factors in children under five living in a malaria-endemic area in Papua, Indonesia
Source: PLoS One. 2025 Nov 4;20(11):e0335643. doi: 10.1371/journal.pone.0335643 (PMC12585096; doi:10.1371/journal.pone.0335643)
Supplement: S1 File — (PDF) [file pone.0335643.s001.pdf]

Standard Operating Procedure: Trichuris PCR in stool samples

1. Objective
To provide an outline of the procedure for Trichuris PCR in stool samples. Trichuris PCR is performed to detect the presence of *Trichuris trichiura* in DNA-extracted stool samples.

2. Equipment
- Laminar flow cabinet
- Pipettes: 1 mL, 200 µL, and 20 µL
- Adjustable pipettes: 0.5–12.5 µL and 10–300 µL
- 96-well flipper rack
- BioRad CFX 384 Real-Time PCR machine
- Plasmid room pipettes: 10 µL and 20 µL

3. Consumables
- Pipette tips: 10 µL and 300 µL
- UNIQUE 10 mL reservoir
- Pipette tips: 20 µL, 200 µL, and 1 mL
- BioRad white-bottom well plates
- BioRad Microseal B adhesive seals
- 5 mL Eppendorf microcentrifuge tubes
- 10 mL Falcon tubes

4. Reagents
- IDT nuclease decontamination solution
- DNase/RNase-free water
- Promega GoTaq 2x Master Mix (GoTaq® qPCR Master Mix-Promega, USA)
- $MgCl_2$ 50 mM
- BSA 20 mg/mL
- Single-use 10 mM primer aliquots (sufficient for 96 wells):
    - TrichurisTsmithFor: 5'-GGC GTA GAG GAG CGA TTT-3'
    - TrichurisTsmithRev: 5'-TAC TAC CCA TCA CAC ATT AGC C-3'
- Single-use 10 mM probe aliquots (sufficient for 96 wells):
    - TrichurisTwillPr-FAM labeled:
      /56-FAM/TT TGC GGG C/ZEN/G AGA ACG GAA ATA TT/3IABkFQ/
- Qiagen Yellow Template Dilution Buffer
- Trichuris PCR plasmid STD1 stock (frozen)

5. Procedure

a. **Control Preparation**
   Plasmid stock is stored at -20°C in the plasmid room. Prepare 20 µL aliquots at a 10^{-2} dilution from the stock using DNase/RNase-free water. These will be used as the standard dilution curve 1 (STD1).

b. **Sample Collection**
   - gDNA from stool samples is stored in the Core Lab. Template addition is performed at the Stool Bench Lab.
   - Samples to be immediately run should be stored at 2–8°C; otherwise, keep at -20°C if DNA extraction was more than a week ago.

- Arrange samples in rows 1–8 on a 96-well flipper rack to facilitate sample addition using a Viaflow Voyager pipette. Store samples at 2–8°C while setting up the PCR machine.

6. Trichuris Singleplex PCR Preparation

a. Perform PCR setup inside the laminar flow cabinet.
b. Preparation of Trichuris PCR reagents in a 5 mL Eppendorf tube is adjusted according to the table below:

   Note: If fewer than 384 wells will be run, adjust the PCR setup according to the number of samples to be run.

Add reagents (dH_2O, $MgCl_2$, BSA, primers, probe, and GoTaq). After all components have been added, vortex. Record the reagent lot numbers in the lab notebook.

Table 1. Composition of Master mix Singleplex real time PCR per reaction

**Trichuris Singleplex PCR Set Up**				**96**
	**Original conc.**	**f.c.**	**Per Rx**	**MM**
Promega GoTaq	2		3,5	**336**
$MgCl_2$	50		0,49	**47.04**
H_2O			0,10	**9.6**
BSA	20mg/ml	2mg/ml	0,70	**67.2**
TrichurisTsmithFor	10	100,00	0,07	**6.72**
TrichurisTsmithRev	10	100,00	0,07	**6.72**
TrichurisTwillPr-FAM	10	100,000	0,07	**6.72**
TOTAL			7	**480**
**Template**	**2**		**Check**	**5**

c. Optional: Add 0.5 µL Qiagen Yellow Template Dilution Buffer to the mastermix to facilitate loading the mastermix into the plate.
d. Transfer the mastermix to the stool bench and pour the mastermix into a UNIQUE 10 reservoir.
e. Clean the ViaFlow Voyager electronic pipette using a nuclease decontamination solution.
f. Turn on the electronic ViaFlow Voyager pipette by pressing the **Run** button, scroll down on the screen, and select the **TrichurisM** program.
g. Attach eight 12.5 µL tips to the pipette and place them into the UNIQUE 10 mL reservoir, then follow the instructions in the pipette program:
   - Press **RUN** to aspirate 12.5 µL of mastermix.
   - Press **RUN**, and 1.0 µL of mastermix will be returned to the reservoir.

- Press **RUN** to dispense 5 μL of mastermix into the well plate (dispense in an A–H direction for efficiency). Continue by pressing **RUN** twice to fully dispense all mastermix into the well.
- Press **RUN** to return the remaining mastermix to the reservoir.
- Repeat until all wells are filled with mastermix. If the mastermix is insufficient, switch to standard pipetting with a multichannel pipette, adding 5 μL in a single dispense.

h. Remove the samples stored at 1-8°C. If condensation is present, homogenize the samples using a microcentrifuge or minifuge.
i. Reuse the ViaFlow Voyager electronic pipette, switching the program to **MULTIPLEX**.
j. Open the sample tubes and add them to each well on the plate, noting the sample positions in the lab notebook.
k. Attach tips to the pipette and follow the program instructions on the screen:
    - Press **RUN** to increase the pipette spacing. Place the pipette tips until they touch the bottom of the tube.
    - Press **RUN**, and the pipette will mix the sample twice.
    - Press **RUN**, and the sample remaining in the tip will be returned to the tube.
    - Press **RUN**, and 4.5 μL of sample will be aspirated.
    - Press **RUN**, and the pipette spacing will decrease to facilitate dispensing into the well.
    - Press **RUN** to dispense 2 μL of sample into the first duplicate well, then press **RUN** again to dispense 2 μL into the second duplicate well.
    - Press **RUN** to expel the remaining sample.
    - Press **EJECT** to discard the tips into the provided waste container.
    - Close the eight sample tubes that have been pipetted and open the next tubes. Repeat the same procedure until all wells are filled.

l. Transfer the plate to the Plasmid Storage Room and retrieve the Trichuris standard plasmid aliquot from the freezer. Add 2 μL of Trichuris STD1 stock to 18 μL of dH_2O and repeat six times for serial dilutions (control samples STD1–7).
m. Add 2 μL (duplicates) to wells according to pre-defined positions (refer to an image or create and document your own layout).
n. Seal the well plate with microseal and ensure it is tightly closed.
o. Transfer the prepared PCR plate to CBCRC for PCR run using the BioRad CFX384. Spin the plate using a spinner before placing it into the PCR machine.

7. Running BioRad CFX384

- Log in to the computer and open BioRad software.
- Choose **REPEAT RUN**, and find **TRICHURIS PCR** in the folder.
- Verify PCR conditions:
    - 95°C for 3 minutes
    - 40 cycles of:
        - 95°C for 10 seconds
        - 61°C for 1 minute
- Click **NEXT**, ensure FAM channel (probe label) is selected.
- Click **NEXT** again to confirm all wells are marked as "unknown" and FAM channel is selected for acquisition.
- Click **RUN**, save the run file, and label it with “Trich”, date, and run number.

8. Post-PCR Analysis

- Open and save the PCR schedule file.
- Copy the complete plate layout and paste it into the corresponding section.
- Copy the sample list and save it as **“sample names for run xxx”**.
- After running is complete:
    - Open the file, go to **GENE EXPRESSION** tab.
    - Select **PLATE SET UP**, then **REPLACE PLATE FILE** with “sample names for run xxx”.
    - In the **QUANTIFICATION** tab, select the FAM channel.
    - Copy Content, Sample names, and Cq values into the PCR data Excel spreadsheet (near multiplex PCR results).
    - Also save the reaction efficiency and R^2 values.

Reference:

CFX Opus 96 Dx, CFX Opus 384 Dx, and CFX Opus Deepwell Dx Real-Time PCR Systems Operation Manual. Manual revision: October 2022 Software revision: 2.3. Available at: https://www.bio-rad.com/sites/default/files/2021-08/10000135538.pdf
