# Supplementary material for: Molecular diagnosis of Trichuris trichiura: Prevalence and associated risk factors in children under five living in a malaria-endemic area in Papua, Indonesia
Source: PLoS One. 2025 Nov 4;20(11):e0335643. doi: 10.1371/journal.pone.0335643 (PMC12585096; doi:10.1371/journal.pone.0335643)
Supplement: S2 File — (PDF) [file pone.0335643.s002.pdf]

## DNA Extraction Procedures

1. Add approximately 150 mg of fecal sample to a ZR BashingBead™ Lysis Tube (0.1 & 0.5 mm) pre-filled with Gotri, then add 750 µl of BashingBead™ Buffer. Secure the tube with parafilm or plastic wrap to prevent leakage. Homogenize using a bead beater at maximum speed for 5 minutes. Centrifuge the tube in a microcentrifuge at 10,000 × g for 1 minute.

2. Transfer up to 400 µl of the supernatant to a Zymo-Spin™ IIIF Filter in a Collection Tube and centrifuge at 8,000 × g for 1 minute. Add 1,200 µl of Genomic Lysis Buffer to the filtrate in the collection tube. Mix well, then transfer the mixture to a Zymo-Spin™ IIC Column in a new Collection Tube. Centrifuge at 10,000 × g for 1 minute. Discard the flow-through, and repeat this step until all filtrate has been processed.

3. Add 200 µl of DNA Pre-Wash Buffer to the IIC Column in a new Collection Tube and centrifuge at 8,000 × g for 1 minute. Then add 500 µl of gDNA Wash Buffer to the IIC Column and centrifuge at 10,000 × g for 1 minute. Transfer the IIC Column to a clean 1.5 ml microcentrifuge tube and add 100 µl of DNA Elution Buffer directly to the column matrix. Centrifuge at 10,000 × g for 30 seconds to elute the DNA.

4. Place a Zymo-Spin™ III-HRC Filter in a clean Collection Tube and add 600 µl of Preparation Solution. Centrifuge at 8,000 × g for 3 minutes. Transfer the eluted DNA to the prepared Zymo-Spin™ III-HRC Filter in a clean 1.5 ml microcentrifuge tube and centrifuge at exactly 16,000 × g for 3 minutes. The filtered DNA is now ready for PCR applications.
