# Supplementary material for: Molecular diagnosis of Trichuris trichiura: Prevalence and associated risk factors in children under five living in a malaria-endemic area in Papua, Indonesia
Source: PLoS One. 2025 Nov 4;20(11):e0335643. doi: 10.1371/journal.pone.0335643 (PMC12585096; doi:10.1371/journal.pone.0335643)
Supplement: S3 Table — (PDF) [file pone.0335643.s003.pdf]

Risk factors for anaemia

Risk Factors	Proportion with anaemia	Univariate Analysis		Multivariate Analysis	
		OR (95% CI)	p	aOR (95% CI)	p
**Age group (years)**					
< 3	19.7 (28/142)	Reference		Reference	
≥ 3	12.8 (5/39)	0.59 (0.21-1.67)	0.327	0.52 (0.15-1.83)	0.309
**Sex**					
Male	17.7 (17/96)	Reference		Reference	
Female	18.8 (16/85)	1.08 (0.51-2.29)	0.846	0.90 (0.36-2.25)	0.818
**Ethnic groups**					
Non Papuan	14.2 (17/120)	Reference		Reference	
Papuan	26.2 (16/61)	2.15 (1.00-4.64)	0.050	1.77 (0.68-4.60)	0.242
**Nutritional status (height for weight)**					
Normal	17.3 (22/127)	Reference		Reference	
Wasting	17.9 (5/28)	1.04 (0.36-3.03)	0.946	1.12 (0.31-4.03)	0.862
Severe wasting	21.7 (5/23)	1.33 (0.44-3.95)	0.613	1.16 (0.28-4.89)	0.839
**Stunting**					
No	15.9 (17/107)	Reference		Reference	
Yes	21.6 (16/74)	1.46 (0.68-3.12)	0.328	1.24 (0.47-3.27)	0.664
**Malaria status**					
Negative	14.1 (23/163)	Reference		Reference	
Positive	55.6 (10/18)	7.61 (2.72-21.29)	0.0001	5.77 (1.70-19.65)	0.005
**Trichuriasis by qPCR**					
No	13.7 (17/124)	Reference		Reference	
Yes	28.1 (16/57)	2.46 (1.14-5.31)	0.023	2.25 (0.89-5.73)	0.089
**Socio-economic status**					
Richest	12.7 (9/71)	Reference		Reference	
Upper-middle	14.5 (9/62)	1.17 (0.43-3.16)	0.757	1.16 (0.40-3.34)	0.785
Middle	6.7 (1/15)	0.49 (0.06-4.21)	0.517	0.26 (0.02-2.75)	0.262
Lower- middle	44.4(12/27)	5.51 (1.96-15.47)	0.001	2.70 (0.84-8.75)	0.097
Lowest	33.3 (2/6)	3.44 (0.55-21.60)	0.187	0.44 (0.03-7.25)	0.568

Co-infection and anaemia

**Infection**	**Anaemia**		**Total**
	**Yes**	**No**	
T. trichiura only	10	39	49
T. trichiura and malaria	6	2	8
**Total**	16	41	57

Of 57 children with trichuriasis by qPCR, the risk of anaemia is significantly increased (75%, 6/8) if co-infected with malaria compared to those with mono *T. trichiura* infection (20.4%, 10/49) with an OR of 11.7 (95%CI, 2.0-67.0), p=0.004.
