# Supplementary material for: Molecular diagnosis of Trichuris trichiura: Prevalence and associated risk factors in children under five living in a malaria-endemic area in Papua, Indonesia
Source: PLoS One. 2025 Nov 4;20(11):e0335643. doi: 10.1371/journal.pone.0335643 (PMC12585096; doi:10.1371/journal.pone.0335643)
Supplement: S4 File — (PDF) [file pone.0335643.s004.pdf]

Discriminant Analysis of Principal Components

Asset that could indicate wealth in the study location was selected based on the previous household survey data and information on locally relevant indicator of wealth (1). We used discriminant analysis of principal components to select assets that best separate SES groups (poorest to richest). The discriminant analysis would maximise the between group variability and minimise within group variability (2). This methods captured heterogeneity in ownership that was not seen using a standard principal component analysis (2).

Result from the Discriminant analysis of Principal Component to construct socio-economic status groupings is shown below:

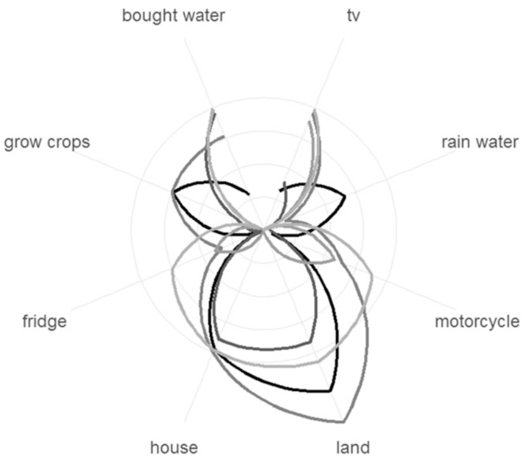

Supplemental Figure 1. Tree plot of ownership by groups from poorest (black) to richest (lightest gray). A discriminant analysis of principal components used to construct these groupings.

References

1. Karyana M, Devine A, Kenangalem E, Burdarm L, Poespoprodjo JR, Vemuri R, et al. Treatment-seeking behaviour and associated costs for malaria in Papua, Indonesia. Malar J. 2016;15(1):536.
2. Vyas S, Kumaranayake L. Constructing socio-economic status indices: how to use principal components analysis. Health Policy Plan. 2006;21(6):459-68.
