# Supplementary material for: Molecular diagnosis of Trichuris trichiura: Prevalence and associated risk factors in children under five living in a malaria-endemic area in Papua, Indonesia
Source: PLoS One. 2025 Nov 4;20(11):e0335643. doi: 10.1371/journal.pone.0335643 (PMC12585096; doi:10.1371/journal.pone.0335643)
Supplement: Attachment — Submitted filename: PLOS Review.docx [file pone.0335643.s006.docx]

*This work provides baseline data for comparison with recent data and efficient use of archived resources.*

*Abstract*

*Methods*

The method of diagnosis of malaria and assessment of anaemia should be included.

*Introduction*

The introduction should include a brief discussion on the interaction between malaria and STH.

*Methods*

Change molecular works to Molecular analysis

*Master mix was prepared at the Parasitology Laboratory, Faculty of Medicine, Public Health and Nursing, Unversitas Gadjah Mada, Yogyakarta.*

Explain why the master mix was prepared in another location and what precautions were taken to avoid contamination and degradation.

*Singleplex qPCR*

*The preparation of controls and generation of a standard curve should be included in the methods and results section.*

*The master mix was prepared based on prior optimization and pipetted at 5 µL into 1.5 mL Eppendorf tubes, followed by the addition of 25 µL of homogenized DNA template.*

This contradicts the information in the supplementary file.

*Assessment of trichuriasis, nutritional status, and socioeconomic status*

*T. trichiura infection (trichuriasis) was defined as positive qPCR results.*

What Ct values were classified as positive?

*Supplementary S1*

Promega GoTaq is used for PCR. Authors should check and name the correct reagent

*Statistical analysis*

*Potential risk factors for trichuriasis included for the analysis were age (< 3 years and >3 years old), sex (male and female), ethnic groups (Papuan and non-Papuan), nutritional status by weight for age (normal, wasting, and severe wasting), stunting (yes/no),* ***malaria (yes/no), anaemia (Hb 10 g/dl)*** *......*

The authors did not describe the methods for detection of malaria infection and assessment of anaemia.

*Results*

*Nearly one-third of the children had low nutritional status (****28.6%,*** *178 51/178) and 41% (74/181) were classified as stunted.*

Check that values match with Table 2. Result presentation pattern should be uniform. Separate the bolded from the bracket.

*Prevalence of T. trichiura infection*

The results should be represented by a table showing both methods of diagnosis.

*The prevalence of T. trichiura infection was 13.8% (25/181) by Kato-Katz microscopy examination and 31.5% (57/181) by qPCR, increasing the prevalence by 17.7%. Of 156 children with negative results by microscopy, 21% (33) had T. trichiura detected by qPCR.*

The figures suggest that only 32 samples were positive by qPCR not 33. Clarify

*There was one sample with T. trichiura detected by microscopy, but not detected by qPCR.*

Was this sample rerun?

*The range of quantification cycle (Cq) value observed in this study was from 24.09 to 37.86.*

Authors should explain the rational of considering Ct of 37.86 as positive.

*Risk factors associated with trichuriasis*

With reference to this earlier statement in methods “*Multiple logistic regression was used to analyse independent risk factors for trichuriasis by entering all significant risk factors with p-value <0.05 in univariate analysis*”,

Confounder are excluded, interactions are missed and borderline predictors are excluded.

The multivariate logistic regression should be rerun with all the risk factors. Same applies to Supplementary S3

*Malaria and trichuriasis coinfection*

Result should be shown in a table for clarity.

*Discussion*

*Line 287 The prevalence of children defined as wasting and stunting*

Change to wasted and stunted

The results from the reanalysis of logistic regression should be incorporated into the discussion.
