# Supplementary material for: Molecular diagnosis of Trichuris trichiura: Prevalence and associated risk factors in children under five living in a malaria-endemic area in Papua, Indonesia
Source: PLoS One. 2025 Nov 4;20(11):e0335643. doi: 10.1371/journal.pone.0335643 (PMC12585096; doi:10.1371/journal.pone.0335643)
Supplement: Attachment — Submitted filename: Dhika et al response to reviewers_v2_21075.docx [file pone.0335643.s007.docx]

**Response to the reviewers' comments:**

**Reviewer #1:**

**1. General Impression**

**This is a well-organized and important study that explores the underreported burden of Trichuris trichiura infection in under-five children using a sensitive molecular diagnostic method (qPCR) in a malaria-endemic region of Papua, Indonesia. The paper is relevant and timely, addressing an important public health gap in low-resource tropical settings. It contributes valuable data on early childhood helminthiasis and its relationship with anaemia and malaria coinfection.**

**2. Major Strengths**
**Use of qPCR: The application of molecular diagnostics strengthens the study's validity and highlights the underestimation of infection prevalence with conventional microscopy.

Age Focus: The focus on children <5 years is crucial as they are often excluded from deworming programs.

Rigorous Methodology: The study is methodically sound with a clear description of the sample collection, molecular techniques, and statistical analyses.

Clinical Relevance: The finding of a significant association between trichuriasis and anaemia, especially in malaria-endemic regions, is highly relevant for integrated disease control policies.**

We thank the reviewer for the positive review.

**3. Weaknesses and Areas for Improvement**

**A. Study Design and Limitations
Cross-sectional nature: The manuscript acknowledges the limitation of causality inference but could benefit from a clearer justification on how the study informs policy despite its observational design.**

Thank you for the suggestion. We have added a line on how the study would inform policy in lines 431-442:

*“Nevertheless, this study offers preliminary evidence on the underrecognized burden of trichuriasis and its risk factors, highlighting the urgent need to implement routine deworming programs for children under five in this setting”.*

**Time lag in sample analysis: There is a 6-year delay between stool sample collection (2013) and qPCR analysis (2019). The authors should elaborate on the impact of this delay on sample integrity, especially regarding DNA degradation, despite proper storage conditions.**

Thank you for pointing this out. We have added a statement on sample integrity associated with long term storage in lines 131-134:

*“Directly frozen stool samples, even without preservatives, remain suitable for qPCR detection (1). Extended freezing preserves DNA integrity, allowing helminth DNA to be effectively recovered during extraction (2).”*

**B. Laboratory and Diagnostic Issues
Coinfections by qPCR: The study only assessed T. trichiura using qPCR. Given that multiple STH infections are prevalent, the absence of multiplex qPCR for Ascaris and hookworms limits a comprehensive parasitological profile.**

**Lack of DNA quantification: The authors acknowledge the absence of DNA quantification due to lack of a spectrophotometer. This affects the ability to assess infection intensity via qPCR, which is a missed opportunity.**

Yes, we acknowledge that due to funding constraints, we were unable to detect other STHs. We would also like to clarify our previous statement regarding the unavailability of a functioning spectrophotometer. After further discussion with the team, we confirmed that the spectrophotometer was functioning properly and that the inability to assess infection intensity with qPCR was due to the DNA concentration measured by NanoDrop did not correlate with Ct values. Accordingly, we have revised the relevant statement in the discussion section. While this represents a limitation, we believe our findings still offer valuable insights into the true burden of *T. trichiura* infections in malaria-endemic areas.

We have acknowledged and revised the limitation in lines 336-338:

*“However, in this study, the DNA concentration measured by NanoDrop (0.3-5.2 ng/ µL) did not show a correlation with the corresponding Ct values. As a result, our findings were interpreted qualitatively”.*

**No validation data: The manuscript should include validation parameters (e.g., sensitivity, specificity, LOD) for the qPCR assay used, possibly referencing the standard curve or internal controls.**

Thank you for the question. We were unable to fully quantify validation parameters because we could not directly determine the baseline DNA concentration of the controls. Instead, we used serially diluted plasmid as a control samples and amplification was performed to generate a standard curve to determine the detection limit (3).

We have included a line to briefly describe the method used in lines 169-177 in the manuscript:

*“A plasmid standard for Trichuris served as the positive control.* *This control was serially diluted from 10^-2^ to 10^-7^ using nuclease-free water, and amplification was performed to generate a standard curve, which was used to determine the detection limit and to assess positive and negative samples* (3).

We have also clarified the Ct cut-off in lines 191-192:

*“T. trichiura infection was defined as a positive qPCR results using a cycle threshold (Ct) cut-off of 40 based on a previously published study using a similar method* (4)*”.*

We would also like to clarify our previous statement regarding the unavailability of a functioning spectrophotometer. After further discussion with the team, we confirmed that the spectrophotometer was functioning properly. Accordingly, we have revised the relevant statement in the discussion section.

We have acknowledged and revised the limitation in lines 336-338:

*“However, in this study, the DNA concentration measured by NanoDrop (0.3-5.2 ng/ µL) did not show a correlation with the corresponding Ct values. As a result, our findings were interpreted qualitatively”.*

**C. Statistical Presentation
Confidence intervals for prevalence: The point prevalence estimates (e.g., 31.5%) would be more robust if accompanied by 95% confidence intervals.**

We have added 95%CIs to prevalence estimates throughout the manuscript.

**Socioeconomic factors: The study used principal component analysis to generate SES quintiles, but the description lacks detail. The authors should briefly mention the input variables used and the rationale for their choice.**

Thank you for the reviewer’s feedback on the need of a more detailed information on the principal component analysis methods used to generate socioeconomic quantiles.

We have revised the wordings to briefly describe the methods used in lines 206-208:

*“Households were classified into SES groups using discriminant analysis of principal components based on asset ownership, then ranked from poorest to richest by both income and expenditure”*.

**D. Interpretation and Discussion
Stunting and wasting: The lack of statistical association between trichuriasis and stunting/wasting is noted, but alternative explanations (e.g., sample size limitation, confounders) are not sufficiently discussed.**

Thank you for pointing this out. We have revised the wording of the relevant paragraph (lines 419-420) to improve clarity:

*“The prevalence of trichuriasis is higher in both children with wasting and stunting, but not statistically significant. The lack of significance may be attributed to the small number of undernourished children in this study. Anaemia, chronic blood loss and impaired intestinal absorption due to inflammation can lead to undernutrition and stunted growth (5-7). However, assessing causality between STH infection and nutritional status is challenging, particularly in a cross-sectional study (8-10). In addition, the impact of malaria and STHs on growth is not straightforward and is affected by recurrent or chronic infections along with other factors (11)”.*

**Anaemia causality direction: The bidirectional relationship between anaemia and helminths is complex. The authors should clarify whether anaemia is interpreted as a cause or consequence of infection—or both.**

Thank you for raising this important point. We have added a statement of the interpretation of anaemia as a potential cause of trichuriasis/STHs in the manuscript (lines 391-394):

*“Iron-deficiency anaemia is known to increase susceptibility to invasive bacterial infections by impairing immune responses (12, 13); however, its association with greater susceptibility to trichuriasis—or STH infections more broadly—remains unclear. In this study, the iron status of the children was not assessed”.*

**E. Ethical and Reporting Standards
Ethics: Ethical approval is adequately addressed. However, the long time gap between sample collection and analysis requires a brief ethical justification or clarification on data use agreements.**

Thank you for pointing this out. We believe that a 7-year gap (2013 to 2019) remains acceptable, as the study posed minimal risk to participants, generated important data, and was reviewed and approved by the ethics committee in 2018.

We also believe that that we have provided sufficient ethical justification in our manuscript (lines 226-236):

***“Additional informed consent was not required, as prior consent from the 2013 household survey included approval for further analysis of stool specimens. Written consent was obtained at the time, including parental consent for children under 14. The ethics committee approved the study and waived the need for new consent”.***

**Funding & Conflict of Interest: The study claims no specific funding. Yet molecular work requires significant resources. A clearer explanation of in-kind contributions from partner institutions (e.g., QIMR) would improve transparency.**

Thank you for pointing this out. We actually have acknowledged the in-kind contribution of the Clinical Tropical Medicine Laboratory, QIMR Berghofer Medical Research Institute, Queensland, Australia in the acknowledgement section in the manuscript (lines 464-469).

*“We are grateful to Ms. Stacey Llewellyn and Dr. James S McCarthy from the Clinical Tropical Medicine Laboratory, QIMR Berghofer Medical Research Institute, Queensland, Australia for their in kind contributions (PCR consumables) and for sharing the SOPs, expertise and experiences in doing the molecular analysis”*

**4. Novelty and Contribution
This is the first known report of T. trichiura prevalence using qPCR in under-five children in Papua, Indonesia. The study adds to the limited literature on preschool STH infections and coinfection risks in high-burden malaria areas. The recommendation to include under-fives in deworming programs is evidence-informed and impactful.**

We thank the reviewer for the positive feedback.

**5. Recommendations for Improvement
Add 95% confidence intervals for prevalence data.**

We have added 95%CIs to prevalence estimates throughout the manuscript.

**Provide more detail on SES scoring method.**

Please also see our response to section C below.

Thank you for the reviewer’s feedback on the need of a more detailed information on the principal component analysis methods used to generate socioeconomic quantiles.

We have revised the wordings to briefly describe the methods used in lines 206-208:

*“Households were classified into SES groups using discriminant analysis of principal components based on asset ownership, then ranked from poorest to richest by both income and expenditure”*.

**Clarify the impact of long-term sample storage on molecular results.**

Please also see our response to comments A above. We have added a statement on sample integrity associated with long term storage in lines 131-134:

*“Directly frozen stool samples, even without preservatives, remain suitable for qPCR detection (1). Extended freezing preserves DNA integrity, allowing helminth DNA to be effectively recovered during extraction (2).”*

**Discuss the lack of multiplex qPCR for other STHs as a limitation.**

Thank you for the suggestion. We have discussed this limitation in lines 427-429 of our manuscript:

*“……Firstly, due to funding limitations, we are unable to present STH coinfections based on qPCR; thus, the true burden of multiple STH infections might be underestimated”.*

**Include information on the diagnostic performance of the qPCR method used.**

We have included a reference and link regarding information on the diagnostic performance of the qPCR method used in Supplementary S1 file:

*CFX Opus 96 Dx, CFX Opus 384 Dx, and CFX Opus Deepwell Dx Real-Time PCR Systems*

*Operation Manual. Manual revision: October 2022 Software revision: 2.3. Available at:* [*https://www.bio-rad.com/sites/default/files/2021-08/10000135538.pdf*](https://www.bio-rad.com/sites/default/files/2021-08/10000135538.pdf)

**Elaborate briefly on implications for national deworming policy—should under-fives be routinely included?**

Please also refer to our response to comments A above.

Thank you for the suggestion. We have added a line on how the study would inform policy in lines 431-442:

*“Nevertheless, this study offers preliminary evidence on the underrecognized burden of trichuriasis and its risk factors, highlighting the urgent need to implement routine deworming programs for children under five in this setting”.*

**Minor Comments**
**Lines 1–2: Revise title to: '...in a malaria-endemic area...' for grammatical correctness.**

We have revised the title accordingly.

**Lines 39–41: Add 95% confidence intervals to prevalence estimates in the abstract for clarity.**

We have added 95%CIs to prevalence estimates throughout the manuscript.

**Lines 47–48: Consider acknowledging limitations of qPCR availability in resource-limited settings.**

Thank you for the suggestion. We acknowledge the importance of the statement and have highlighted this in the conclusion section of the manuscript (lines 322-325).

**Lines 61–63: Rephrase to avoid redundancy: use 'diagnostic methods relying on direct microscopy, which has low sensitivity.'**

We have revised the wordings accordingly in lines 87-88.

**Lines 70–72: Clarify study aims: '...estimate the prevalence using qPCR and identify associated risk factors...'**

We have modified the wordings to clarify the study aims in lines 96-98.

**Lines 86–89: Clarify sampling process for 181 children from 629 enrolled to explain inclusion criteria.**

Thank you for pointing this out. Since we were using archived samples, our inclusion criteria were based on the availability of stool samples and the associated clinical data, as stated in the current manuscript (lines 124-125):

*“Children aged <5 years old with complete clinical data and stool samples were included in the analysis”.*

**Line 92: Clarify 'without preservatives' by explaining sample storage conditions more explicitly.**

We have clarified the stool storage conditions in lines 128-129:

*“The remaining stool samples were stored without further processing in a -80^0^C freezer at the research laboratory in Timika”*

**Lines 103–105: Indicate whether internal controls were used to check for DNA degradation.**

We did not use internal control to check for DNA degradation, based on the assumption that helminth DNA integrity was preserved during the storage in -80 freezer (1) (2). Please refer to our responses addressing concerns on sample integrity above.

**Line 120: Mention the method used to quantify plasmid concentration for standard curve accuracy.**

We used serially diluted plasmid standard to generate a standard curve and have acknowledged the limitations of our methods in the discussion section.

We have included a line to briefly describe the method used in lines 169-177 in the manuscript:

*“A plasmid standard for Trichuris served as the positive control.* *This control was serially diluted from 10^-2^ to 10^-7^ using nuclease-free water, and amplification was performed to generate a standard curve, which was used to determine the detection limit and to assess positive and negative samples* (3)”.

We have also acknowledged the limitation in lines 336-338:

*“However, in this study, the DNA concentration measured by NanoDrop (0.3-5.2 ng/ µL) did not show a correlation with the corresponding Ct values. As a result, our findings were interpreted qualitatively”.*

**Line 173: Ensure figure is embedded with correct caption below and properly labeled.**

We have reviewed the associated figure caption and we confirm that it is properly labelled.

**Lines 186–191: Include 95% CI and test statistical significance of increased detection by qPCR.**

We have added 95%CI and the p value of the increased detection by qPCR in line 258.

**Line 199: Clarify detection method for coinfections—were *Ascaris* and hookworm microscopy-only?**

We have clarified the method of detection of *Ascaris lumbricoides* and hookworm infection in lines 274-275.

**Table 2: Add confidence intervals where appropriate; specify handling of missing data.**

Thank you for the suggestion. However, we presented table 2 to describe subject’s characteristics. We think that the 95%CIs of the study outcome prevalence was better presented in the manuscript text.

The 3 subjects with missing weight data were excluded from the analysis. We have included a line to clarify the handling of missing data in the table’s footnote (line 253):

*“*3 missing weight data were excluded from the analysis”.*

**Table 3: Include subgroup sample sizes and clarify total N used for each category.**

Thank you for the suggestion. The subgroup sample sizes and N used for each category are presented in n/valid cases in the second column (*T. trichiura* infections by PCR).

**Lines 240–241: Acknowledge potential impact of DNA degradation due to long-term storage.**

Thank you for the suggestion. We have added a line acknowledging the potential impact of long term sample storage in lines 338-340:

*“In addition, possible DNA degradation due to long term sample storage may lead to the underestimation of the true prevalence and may hinder the assessment of infection intensity (1, 2).”*

**Line 248: Use consistent age group terminology (either '<3 and >3' or specific year ranges).**

We have corrected the age group terminology to ≤ 3 years old and >3 years old in lines 346-347 for consistency.

**Line 259: Soften speculative language unless supported by qualitative data.**

Thank you for pointing this out. We have toned down the statement to better reflect the nature of the study. Please see lines 372-373:

*“This might indicate gaps in knowledge about STHs transmission and related hygiene and sanitation practices in the study households”.*

**Lines 295–299: Mention lack of multiplex qPCR for other STHs as a limitation.**

Thank you for the suggestion. However, we believe that our current statement in the manuscript have clearly outlined the reviewer’s suggestion (lines 427-429):

*“Firstly, due to funding limitations, we are unable to present STH coinfections based on qPCR; thus, the true burden of multiple STH infections might be underestimated”.*

**Lines 328–442: Ensure reference formatting is consistent and check for duplicate citations.
Throughout: Unify terminology (e.g., 'trichuriasis' vs '*T. trichiura* infection'), units (g/dL), and italicization of species names.**

Thank you for pointing this out. We have corrected the inconsistency of units (g/dL) and ensuring species names are italicised throughout the manuscript. On the other hand, we used the term trichuriasis with *T. trichiura* infections interchangeably for stylistic variation. We have mentioned both terms in the first sentence of introduction (line 82). We hope that this is acceptable.

**Reviewer #2:**

**This work provides baseline data for comparison with recent data and efficient use of archived resources.**

**Abstract**

**Methods
The method of diagnosis of malaria and assessment of anaemia should be included.**

We have added a line to briefly describe the methods used for malaria diagnosis and Hb measurement in the manuscript (lines 134-136):

*“Peripheral parasitaemia was assessed by two trained microscopists and haemoglobin concentration was measured using an electronic Coulter counter (Coulter JTTM, USA)*. *The methods are described in detail elsewhere* (14)*”*

**Introduction
The introduction should include a brief discussion on the interaction between malaria and STH.**

We have added a line on the malaria and STHs interactions in the introduction section (lines 90-91):

*“Although remains inconclusive, another important factor is that STHs infection may increase susceptibility to P. falciparum infection”.*

**Methods
Change molecular works to Molecular analysis**

We have changed the term “molecular works” to “molecular analysis” in the manuscript.

**Master mix was prepared at the Parasitology Laboratory, Faculty of Medicine, Public Health and Nursing, Unversitas Gadjah Mada, Yogyakarta.
Explain why the master mix was prepared in another location and what precautions were taken to avoid contamination and degradation.**

Thank you for the question. We acknowledge that we don’t have mixing room close to the qPCR machine. We have added a line to describe our transfer procedures from Parasitology Lab to Biochemistry lab (lines 148-151):

*“The PCR plates to be run were sealed tightly and placed in an ice box to maintain optimal DNA conditions and further reduce the risk of contamination during the transport from the Parasitology Laboratory to the Biochemistry Laboratory”.*

**Singleplex qPCR
The preparation of controls and generation of a standard curve should be included in the methods and results section.**

We have included the controls preparation and generation of standard curve in the manuscript (lines 169-177):

*“A plasmid standard for Trichuris served as the positive control.* *This control was serially diluted from 10^-2^ to 10^-7^ using nuclease-free water, and amplification was performed to generate a standard curve, which was used to determine the detection limit and to assess positive and negative samples* (3)”.

**The master mix was prepared based on prior optimization and pipetted at 5 µL into 1.5 mL Eppendorf tubes, followed by the addition of 25 µL of homogenized DNA template.
This contradicts the information in the supplementary file**.

Thank you for pointing this out. We have corrected the amount of DNA template from 25 µL to 2 µL in the manuscript (lines 178-180).

**Assessment of trichuriasis, nutritional status, and socioeconomic status
T. trichiura infection (trichuriasis) was defined as positive qPCR results.
What Ct values were classified as positive?**

We have clarified the Ct values that were classified as positive in the manuscript:

Lines 191-192:

*“T. trichiura infection was defined as a positive qPCR results using a cycle threshold (Ct) cut-off of 40 based on a previously published study using a similar method* (4)*”.*

Lines 260-265:

*The range of quantification cycle/cycle threshold (Ct) value observed in this study was from 24.09 to 37.86 and considered as positive for T. trichiura infection* (1, 4)”.

**Supplementary S1
Promega GoTaq is used for PCR. Authors should check and name the correct reagent.**

We have added the brand name of the reagent in the supplementary file 1: GoTaq® qPCR Master Mix (Promega, USA).

**Statistical analysis
Potential risk factors for trichuriasis included for the analysis were age (< 3 years and >3 years old), sex (male and female), ethnic groups (Papuan and non-Papuan), nutritional status by weight for age (normal, wasting, and severe wasting), stunting (yes/no), malaria (yes/no), anaemia (Hb 10 g/dl) ......
The authors did not describe the methods for detection of malaria infection and assessment of anaemia.**

We have added a line to briefly describe the methods used for malaria diagnosis and Hb measurement in the manuscript (lines 134-136):

*“Peripheral parasitaemia was assessed by two trained microscopists and haemoglobin concentration was measured using an electronic Coulter counter (Coulter JTTM, USA)*. *The methods are described in detail elsewhere* (14)*”*

**Results**
**Nearly one-third of the children had low nutritional status (28.6%, 178 51/178) and 41% (74/181) were classified as stunted. Check that values match with Table 2. Result presentation pattern should be uniform.**

The low nutritional status was based on height for weight measurements. As there were three missing weight data points (from 181 children), the denominator was 178. These values are consistent with those presented in Table 2.

**Separate the bolded from the bracket.**

We have revised accordingly.

**Prevalence of T. trichiura infection
The results should be represented by a table showing both methods of diagnosis.
The prevalence of T. trichiura infection was 13.8% (25/181) by Kato-Katz microscopy examination and 31.5% (57/181) by qPCR, increasing the prevalence by 17.7%. Of 156 children with negative results by microscopy, 21% (33) had T. trichiura detected by qPCR.
The figures suggest that only 32 samples were positive by qPCR not 33. Clarify.**

Yes, the samples that were positive by qPCR was 33. There was one Kato Katz positive but with negative qPCR.

The table was inserted in line 267 of the manuscript:

| **Kato Katz** | **qPCR** | | **Total** |
| --- | --- | --- | --- |
|  | Negative | Positive |  |
| Negative | 123 | 33 | 156 |
| Positive | 1 | 24 | 25 |
| **Total** | 124 | 57 | 181 |

**There was one sample with T. trichiura detected by microscopy, but not detected by qPCR. Was this sample rerun?**

Yes, the sample was rerun and still not detected by qPCR. We have added a line in the manuscript explaining this (lines 259-260):

*“There was one sample with T. trichiura detected by microscopy, but not detected by qPCR even after a rerun (Table 3)”.*

**The range of quantification cycle (Cq) value observed in this study was from 24.09 to 37.86. Authors should explain the rational of considering Ct of 37.86 as positive**.

We have clarified the Ct values that were classified as positive in the manuscript:

Lines 191-192:

*“T. trichiura infection was defined as a positive qPCR results using a cycle threshold (Ct) cut-off of 40 based on a previously published study using a similar method* (4)*”.*

**Risk factors associated with trichuriasis
With reference to this earlier statement in methods “Multiple logistic regression was used to analyse independent risk factors for trichuriasis by entering all significant risk factors with p-value <0.05 in univariate analysis”,
Confounder are excluded, interactions are missed and borderline predictors are excluded.
The multivariate logistic regression should be rerun with all the risk factors. Same applies to Supplementary S3**

We thank the reviewer for the suggestion. We have rerun the multivariable analysis by including all potential risk factors in the models. We have revised table 2 and Supplementary S3 table accordingly.

**Malaria and trichuriasis coinfection
Result should be shown in a table for clarity.**

Thank you for the suggestion. As the numbers are quite small and to keep the main manuscript concise, we have included a table on malaria and trichuriasis co-infection in Supplementary File 3.

| **Infection** | **Anaemia** | | **Total** |
| --- | --- | --- | --- |
|  | Yes | No |  |
| *T. trichiura* only | 10 | 39 | 49 |
| *T. trichiura* and malaria | 6 | 2 | 8 |
| **Total** | 16 | 41 | 57 |

**Discussion
Line 287 The prevalence of children defined as wasting and stunting, change to wasted and stunted.**

We have revised the words accordingly.

**The results from the reanalysis of logistic regression should be incorporated into the discussion.**

After reanalysis, we found that only age >3 years old was an independent risk factors for trichuriasis, whereas anaemia was only significant in univariable analysis, but not in multivariable analysis. We have revised the result (lines 281-282) and discussion section (lines 387-390) according to the new findings:

Lines 281-282:

*………”However after adjusting for other risk factors the association was no longer statistically significant (aOR 2.29 [0.92-5.70], p=0.076)”.*

Lines 387-390:

*……”Interestingly, although not an independent risk factor, our study showed that children with anaemia (Hb≤10 g/dl) living in a malaria-endemic area were two times more likely to have trichuriasis compared to those with Hb greater than 10 g/dl (OR 2.46; 95%CI, 1.14-5.31, p=0.020)”.*

**References:**

1.	Kaisar MMM, Brienen EAT, Djuardi Y, Sartono E, Yazdanbakhsh M, Verweij JJ, et al. Improved diagnosis of Trichuris trichiura by using a bead-beating procedure on ethanol preserved stool samples prior to DNA isolation and the performance of multiplex real-time PCR for intestinal parasites. Parasitology. 2017;144(7):965-74.

2.	Azzopardi KI, Hardy M, Baker C, Bonnici R, Llewellyn S, McCarthy JS, et al. Detection of six soil-transmitted helminths in human stool by qPCR- a systematic workflow. PLoS One. 2021;16(9):e0258039.

3.	Arya M, Shergill IS, Williamson M, Gommersall L, Arya N, Patel HR. Basic principles of real-time quantitative PCR. Expert Rev Mol Diagn. 2005;5(2):209-19.

4.	Kridaningsih TN, Sukmana DJ, Mufidah H, Diptyanusa A, Kusumasari RA, Burdam FH, et al. Epidemiology and risk factors of Strongyloides stercoralis infection in Papua, Indonesia: a molecular diagnostic study. Acta Trop. 2020;209:105575.

5.	Jourdan PM, Lamberton PHL, Fenwick A, Addiss DG. Soil-transmitted helminth infections. Lancet. 2018;391(10117):252-65.

6.	Albonico M, Allen H, Chitsulo L, Engels D, Gabrielli AF, Savioli L. Controlling soil-transmitted helminthiasis in pre-school-age children through preventive chemotherapy. PLoS Negl Trop Dis. 2008;2(3):e126.

7.	Else KJ, Keiser J, Holland CV, Grencis RK, Sattelle DB, Fujiwara RT, et al. Whipworm and roundworm infections. Nat Rev Dis Primers. 2020;6(1):44.

8.	Fauziah N, Aviani JK, Agrianfanny YN, Fatimah SN. Intestinal Parasitic Infection and Nutritional Status in Children under Five Years Old: A Systematic Review. Trop Med Infect Dis. 2022;7(11).

9.	Yoseph A, Beyene H. The high prevalence of intestinal parasitic infections is associated with stunting among children aged 6-59 months in Boricha Woreda, Southern Ethiopia: a cross-sectional study. BMC Public Health. 2020;20(1):1270.

10.	Raj E, Calvo-Urbano B, Heffernan C, Halder J, Webster JP. Systematic review to evaluate a potential association between helminth infection and physical stunting in children. Parasites & vectors. 2022;15(1):135.

11.	Danso F, Appiah MA. Prevalence and associated factors influencing stunting and wasting among children of ages 1 to 5 years in Nkwanta South Municipality, Ghana. Nutrition. 2023;110:111996.

12.	Abuga KM, Nairz M, MacLennan CA, Atkinson SH. Severe anaemia, iron deficiency, and susceptibility to invasive bacterial infections. Wellcome Open Res. 2023;8:48.

13.	Jonker FA, Boele van Hensbroek M. Anaemia, iron deficiency and susceptibility to infections. The Journal of infection. 2014;69 Suppl 1:S23-7.

14.	Burdam FH, Hakimi M, Thio F, Kenangalem E, Indrawanti R, Noviyanti R, et al. Asymptomatic Vivax and Falciparum Parasitaemia with Helminth Co-Infection: Major Risk Factors for Anaemia in Early Life. PLoS One. 2016;11(8):e0160917.
